# Prevalence of malnutrition and associated risk factors among adult visceral leishmaniasis patients in Northwest Ethiopia: a cross sectional study

**DOI:** 10.1186/1756-0500-7-75

**Published:** 2014-02-04

**Authors:** Bewketu Mengesha, Mengistu Endris, Yegnasew Takele, Kalehiwot Mekonnen, Takele Tadesse, Amsalu Feleke, Ermias Diro

**Affiliations:** 1 Leishmaniasis Research and Treatment Center, University of Gondar, Gondar, Ethiopia; 2 Institute of Public Health, University of Gondar, Gondar, Ethiopia; 3 Department of Medical Microbiology, University of Gondar, Gondar, Ethiopia; 4 Department of Internal Medicine, University of Gondar, Gondar, Ethiopia; 5 Institute of Tropical Medicine, Antwerp, Belgium

**Keywords:** Anemia, HIV, Intestinal parasites, Malnutrition, Visceral leishmaniasis

## Abstract

**Background:**

Visceral leishmaniasis (VL) causes considerable morbidity and mortality in Ethiopia. Data on the prevalence and associated risk factors on malnutrition among VL patients in Ethiopia are scarce. This study aimed to assess the prevalence of malnutrition and its associated risk factor among VL patients in Northwest Ethiopia.

**Methods:**

An institution-based cross-sectional study was conducted from June to September 2012 at four leishmaniasis treatment sites in Northwest Ethiopia. Four hundred and three adult VL patients were enrolled in the study. Malnutrition was defined as a body mass index (BMI) ≤ 18.5 kg/m2. The data collected from the VL patients included sex, age, residence, occupation, weight, height, laboratory results (HIV, hemoglobin, intestinal parasites). Multivariate logistic regression model was used to determine the strength of association between malnutrition and associated risk factors.

**Results:**

Among 403 adult VL patients 385 (95.5%) were malnourished. Twenty eight percent (n = 113), 30.3% (n = 122), and 37.2% (n = 150) were mildly, moderately and severely malnourished, respectively. The prevalence of intestinal parasitic infection was 47.6% (n = 192) and it was associated with malnutrition (P = 0.01). The prevalence of VL-HIV co-infection was 10.4% (n = 42). Hook worm, *Giardia intestinalis* and *Ascaris lumbircoides* were the leading prevalent intestinal parasites. Factors such as age, sex, residence, occupation, HIV status and anemia were not associated with severe malnutrition.

**Conclusions:**

The prevalence of malnutrition in VL patients was very high and it was associated with intestinal parasitic infections. Therefore, screening of severely malnourished VL patients for intestinal parasitic infections during admission is recommended.

## Background

Visceral leishmaniasis (VL) is one of the most neglected infectious diseases [1]. It affects largely the poorest of the poor, mainly in developing countries including Ethiopia [2]. VL is 100% fatal if left untreated within two years [3]. Ethiopia is estimated to have the second highest burden in terms of VL among the Sub-Saharan African countries [4]. The Northwest Ethiopia has known endemic foci namely Metema, Humera,Wolkait, Shiraro and Libo/Fogera [5].

Malnutrition is one of the risk factors for the development of VL [6-8]. Malnutrition, HIV, intestinal parasitic infections and anemia play a role in the expression of clinically overt VL disease in the endemic areas [9].

The nutritional status of the host may be impaired by the increased nutrient demands of the intestinal parasite itself or by specific actions such as blocking the absorbing surface of the mucosa by adult worms like *Ascaris lumbricoides*, ongoing blood loss by hookworm infections and/or schistosomiasis [10]. Intestinal parasitoses can suppress cell mediated immune responses and appears to predispose to the development of progressive VL in some patients [11].

According to the 2011 Ethiopian Demographic and Health Survey, the prevalence of overall malnutrition and moderately/severely malnutrition among Ethiopian men aged 15–59 years was 36.6% and 13.4%, respectively. In the same report women age 15–49 26.9% were malnourished in which 9.1% moderately/severely malnourished [12].

Intestinal parasitic infections are among the major public health problems in sub-Saharan Africa, including Ethiopia. One third of Ethiopians are infected with ascariasis, one quarter is infected with trichuriasis and one in eight Ethiopians lives with hookworm [4].

Even though VL had remained endemic in Ethiopia since at least 1942, the prevalence and association of malnutrition amongst VL patients were not well known in the region. This study aimed to determine the prevalence of malnutrition and associated risk factors among patients with VL in Northwest Ethiopia. This will allow improved identification and therefore management of malnutrition in patients with VL, which may in turn lead to better outcomes.

## Methods

### Study setting

An institution based cross-sectional study was conducted from June to September 2012. All patients age ≥18 years and non pregnant women (n = 403) diagnosed with VL at four leishmaniasis treatment centers between June to September 2012 were included in this study. The treatment centers were Gondar Leishmaniasis research and treatment center, Kahsay-Abera hospital, Metema hospital and Addis Zemen health center.

### Data collection, procedures and tools

An interview guided by pre-tested structured questionnaire was used to collect the socio-demography and behavioral determinants of the VL patients.

### Nutritional status assessment

The nutritional status of the patients was determined using body mass index (BMI). The weights of the patients were measured with minimal clothing in kilograms using appropriately calibrated weight scale of 120 kg capacity (Health scale, China). The height of each patient was measured using calibrated anthropometric steel rod measuring device. BMI was calculated by dividing weight (in kg) by height (in m^2^) [13].

### Stool specimen collection and diagnosis

A single fresh stool specimen was collected from VL patients using clean, dry, leak proof and wide mouthed container. About 20 mg of stool was taken and processed using direct saline wet mount to diagnose intestinal parasite/s. One gram of stool was also preserved by 10% formalin and diagnosed for intestinal parasites using formol-ether concentration technique [14]. In all cases the samples were processed and read by two experienced laboratory technologists. In case when the results were discordant, a third expert was used.

### Hemoglobin and HIV status determination

Two milliliters of whole blood from each VL patient were collected by EDTA tube. Fresh whole blood sample was used to determine hemoglobin level of VL patients using hematological analyzer (Beckman^®^ Coulter A^c^T diff™, Beckman^®^ Coulter Inc, 2003, USA) machine.

Rapid test for HIV (1/2) antibody in plasma was performed following the current Ethiopian national HIV diagnostic algorithm [15]. The algorithm includes KHB (Shanghai kehua Bio-engineering CO-Ltd, Shanghai, China) as a primary test and HIV 1/2 STAT-PAK™ (Chembio HIV1/2, Medford, New york, USA) as a secondary test and Uni-Gold™ (Trinity Biotech PLC, Bray, Ireland) as a tie breaker.

### Definitions

#### Case definition of VL

Symptomatic patients who had history of intermittent fever for more than two weeks, spleenomegaly +/- hepatomegaly, and low hemoglobin level (<11 mg/dL) presented at the health institutes and/or then serologically positive for Recombinant Kinesine-39 test. Parasitologically confirmed patients with tissue aspirate (bone marrow, spleen or lymph node) for amastigotes were also included in the study [16].

### Nutritional status classification

The nutritional status definitions were following the WHO [13]. The nutritional status was also defined as mild, moderate, severe and normal when the individual had BMI of <16, ≥16- < 17, ≥17- < 18.5 and ≥18.5, respectively.

### Anemia status

Classification of anemia was using the level of hemoglobin in g/dl [17]. Hemoglobin status between 12–16 g/dl and 13–17 g/dl was considered as normal for female and male patients, respectively. Mild anemia for females and male patients was ranged between 11–11.9 g/dl and 11–12.9 g/dl hemoglobin, respectively. For both sexes with hemoglobin level of 8–10.9 g/dl and <8 was considered as moderately anemic and severely anemic, respectively.

### Data processing and analysis

Data were entered in to EPI-info version 2000, then were rechecked and transferred for analysis to Statistical Package for Social Science (SPSS) version 20 statistical software. Analytical and descriptive statistical test techniques were utilized; bivariate and multivariate analyses were utilized. Variables with P ≤ 0.2 in univariate were tested in multivariate logistics model. P-values less than 0.05 were considered statistically significant.

### Ethical considerations

The study protocol was reviewed and approved by research and ethics committee of Institute of Public Health, University of Gondar. Informed written consent was obtained from each patient. All the laboratory results were communicated to the treating physicians for better management of patients. All the information obtained from the study was kept confidential.

## Results

### Characteristics of the patients

A total of 403 patients participated in the study. The descriptive characteristics of the enrolled patients are shown in Table 1. The VL-HIV co-infection rate was (n = 42; 10.4%) (Table 2).

**Table 1:** Characteristics of VL patients and their malnutrition status in Northwest Ethiopia, 2012

**Characteristics**	**Total examined**	**Severely malnourished**		**COR (95% CI)**	**P-values**
		**Yes**	**No**		
Sex					
Male	397 (98.5)*	149 (37.5)	248 (62.5)	3.00 (0.34 – 68.61)	0.41#
Female	6 (1.5)	1 (16.7)	5 (83.3)	1.00	
Age					
18-27	274 (68.0)	98 (35.8)	176 (64.2)	1.00	0.46
28-37	104 (25.8)	40 (38.5)	64 (61.5)	1.12 (0.69 – 1.84)	
>37	25 (6.2)	12 (48.0)	13 (52.0)	1.66 (0.68 – 4.05)	
Occupation					
Daily laborer	378 (93.8)	140 (37.0)	238 (63.0)	0.88 (0.36 – 2.18)	0.06
Non-daily laborers۩	25 (6.2)	10 (40.0)	15 (60.0)	1.00	
Educational status					
Illiterate	294 (73.0)	105 (35.7)	189 (64.3)	0.79 (0.49 – 1.27)	0.30
Literate	109 (27.0)	45 (41.3)	64 (58.7)	1.00	
Residence					
Rural	363 (90.1)	135 (37.2)	228 (62.8)	0.99 (0.48 – 2.04)	0.51
Urban	40 (9.9)	15 (37.5)	25 (62.5)	1.00	
**Over all**	**403 (100)**	**150 (37.2)**	**253 (62.8)**		

*Figures in parenthesis indicate percentage, ۩Governmental employee = 6, students = 8 and merchants = 11, # Fisher exact.

**Table 2:** Bivariate and multivariate analysis of possible risk factors with malnutrition among patients with VL in Northwest Ethiopia, 2012

**Risk factors**	**Total**	**Severely Malnourished**		**COR (95% CI)**	**AOR (95%CI)**	**P-values**
		**Yes**	**No**			
Eating breakfast						
Always	127 (31.5)*	97 (35.1)	179 (64.9)	1.00	1.00	0.07
Sometimes	276 (68.5)	53 (41.7)	74 (58.3)	1.32 (0.84 – 2.08)	1.56 (0.96 – 2.51)	
Domestic animal						
Present	218 (54.1)	85 (39.0)	133 (61.0)	1.00	1.00	0.48
Absent	185 (45.9)	65 (35.1)	120 (64.9)	0.85 (0.55 – 1.30)	0.99 (0.59 – 1.65)	
Private farm						
Present	168 (41.7)	70 (41.7)	98 (58.3)	1.00	1.00	0.14
Absent	235 (58.3)	80 (34.0)	155 (66.0)	0.72 (0.47 – 1.11)	0.65 (0.38 – 1.11)	
Family size						
≤5	212 (52.6)	79 (37.3)	133 (62.7)	1.00	1.00	0.98#
6-10	185 (45.9)	69 (37.3)	116 (62.7)	1.00 (0.65 – 1.54)	1.12 (0.18 – 6.67)	
≥11	6 (1.5)	2 (33.3)	4 (66.7)	0.84 (0.10 – 5.50)	1.19 (0.21 – 7.05)	
Anemia						
Mild	87 (21.6)	31 (35.6)	56 (64.4)	1.00	1.00	0.42
Moderate	205 (50.9)	72 (35.1)	133 (64.9)	0.98 (0.56 – 1.71)	1.38 (0.76 – 2.52)	
Severe	111 (27.5)	47 (42.3)	64 (57.7)	1.33 (0.71 – 2.47)	1.43 (0.88 – 2.32)	
HIV Status						
Negative	361 (89.6)	130 (36.0)	231 (64.0)	1.00	1.00	0.19
Positive	42 (10.4)	20 (47.6)	22 (52.4)	1.62 (0.81 – 3.21)	1.67 (0.34 – 3.32)	
Intestinal parasites						
Absent	211 (52.4)	50 (23.7)	161 (76.3)	1.00	1.00	0.00
Present	192 (47.6)	100 (52.1)	92 (47.9)	3.50 (2.24 – 5.48)	3.01 (2.20 – 5.11)	

*Figures in parenthesis indicate percentage, # Fisher exact.

### Prevalence of malnutrition

Among the 403 VL patients participated in the study 385(95.5%) were malnourished. Of the malnourished 113(29.3%), 122(31.7%) and 150 (39.0%) were mildly, moderately and severely malnourished, respectively. The prevalence of severe malnutrition among VL-HIV co-infected and HIV negative VL was 47.6% and 36%, respectively (P > 0.05) (Table 2).

### Risk factor

Presence of intestinal parasitic infection was statistically associated with severe malnutrition among VL patients (P < 0.001). However, factors such as age, sex, educational status, residence and occupation were not associated with malnutrition (Table 1). Family size, anemia, HIV status were also not associated with malnutrition (Table 2).

One hundred and ninety two (47.6%) patients were infected with one or more intestinal parasites. Of the nine identified intestinal parasites Hook worm 85(21.1%) was the leading followed by, *Giardia intestinalis* 47(11.7%) and *Ascaris lumbricoides* 41(10.2%). The other intestinal parasites recovered from the stool samples of both malnourished and normal VL patients are presented in Table 3.

**Table 3:** Intestinal parasites among adult VL patients in Northwest Ethiopia, 2012

**Intestinal parasites**	**Total Number (%)**	**Severely malnourished**	
		**Yes Number (%)**	**No Number (%)**
**Protozoas**			
*Entamoeba histolytica/dispar*	40 (9.9)	31 (77.5)	9 (22.5)
*Giardia intestinalis*	47 (11.7)	34 (72.3)	13 (27.7)
**Helminthes**			
*Ascaris lumbricoides*	41 (10.2)	29 (70.7)	12 (29.3)
Hook worm	85 (21.1)	50 (58.8)	35 (41.2)
*Hymenolopsis nana*	3 (0.7)	2 (66.7)	1 (33.3)
*Schistosoma mansoni*	4 (1.0)	4 (100)	0 (0.0)
*Strongyloide stercolaris*	23 (5.7)	14 (60.9)	9 (39.1)
*Tanea* species	4 (1.0)	4 (100)	0 (0.0)
*Trichuris trichiura*	4 (1.0)	3 (75.0)	1 (25.0)

The rate multiple intestinal parasitic infection was 48 (11.9%) of which all were severely malnourished. The frequently detected multiple intestinal parasites recovered from severely malnourished patients were *Ascaris lumbricoides* and Hook worm (n = 13) followed by *Entamoeba histolytica/dispar* and *Giardia intestinalis* (n = 7)*,* and *Entamoeba histolytica/dispar* and Hook worm (n = 5) (Table 4).

**Table 4:** Multiple parasitic infections among severely malnourished adult VL patients in Northwest Ethiopia, 2012 (N = 48)

**Multiple parasitic infections**	**Frequency**
*Ascaris lumbricoides* + Hook worm	13
*Entamoeba histolytica/dispar + Giardia intestinalis*	7
*Entamoeba histolytica/dispar* + Hook worm	5
Hook worm + *Strongyloide stercolaris*	4
*Giardia intestinalis* + Hook worm	4
*Giardia intestinalis + Ascaris lumbricoides*	4
*Giardia intestinalis + Strongyloide stercolaris*	4
*Schistosoma mansoni + Entamoeba histolytica/dispar*	2
*Ascaris lumbricoides + Strongyloide stercolaris*	1
*Hymenolopsis nana + Entamoeba histolytica/dispar*	1
*Giardia intestinalis +* Hook worm + *Entamoeba histolytica/dispar*	2
*Giardia intestinalis +* Hook worm + *Strongyloide stercolaris*	1

## Discussion

The overall malnutrition among adult patients with VL was 95.5%. Among 403 VL patients 150 (37.2%) patients were severely malnourished. This result was consistent with the previous study among VL patients in Ethiopia 39.3% [18].

VL was commonly seen among male participants, 397(98.5 %) than females 6(1.5%). This might be due to the increased tendency of the male participants in rural to spend most of the time outside home for agricultural activities where they might be bitten by sand flies [19].

The prevalence of severe malnutrition among VL-HIV co-infected patients (47.6%) was higher than HIV negative VL patients (36%) though statistically not significant (P > 0.05).

In the present study, 100% of the VL patients were found to be anemic. Similarly a study done in Uganda revealed that 96% of VL patients presented with anemia [20]. According to the 2011 Ethiopian demographic and health survey 17% and 11% of women and men were severely anemic, respectively [12]. The observed prevalence in patients with VL was very high. The severe malnutrition status among mildly anemic, moderately anemic and severely anemic VL patients was 35.6%, 35.1% and 42.3%, respectively. There is overlap of malaria and VL in lowland of the Northwest Ethiopia. Repeated attack of malaria and loss of appetite might contribute to the high prevalence of anemia and malnutrition in VL patients in the area.

Intestinal parasitic infection was found to be significantly associated with severe malnutrition among VL patients (P < 0.01). Patients with VL who had one or more intestinal parasitic infection were three times more likely to be severely malnourished than patients with no intestinal parasites [AOR: 3.01 (2.20 – 5.11)]. In this study 192 (47.6%) of VL patients were infected with one or more intestinal parasites. Of whom more than half (n = 100; 52.1%) were severely malnourished. Forty eight VL patients had multiple intestinal parasitic infections; all were severely malnourished. Malnutrition due to intestinal parasitoses might be due to nutrient demand of parasite itself or blocking of the absorbing surface of the mucosa by adult worms, and ongoing blood loss by hookworm infections and/or schistosomiasis [10].

VL can be a risk factor for malnutrition and can lead to low BMI. On the other hand, malnutrition can affect the immune system of the host, making people vulnerable to infections. Malnutrition can make a person susceptible to infection and infection also contributes to malnutrition, which causes a vicious cycle [21].

### Limitation of the study

We use only BMI which depends upon weight and the square of height, it ignores basic scaling laws whereby mass increases to the 3rd power of linear dimensions.

## Conclusions

The prevalence of severe malnutrition and intestinal parasitic infection among adult VL patients was found high and associated. Therefore, screening of severely malnourished VL patients for intestinal parasitic infections is recommended. Possibilities of synchronized nutritional rehabilitation and universal deworming programs with VL treatment should be looked for.

## Abbreviations

BMI: Body mass index; HIV: Human immuno-deficiency virus; OR: Odds ratio; VL: Visceral leishmaniasis.

## Competing interests

The authors declare that they have no competing interests.

## Authors’ contributions

BM, ME, YT conceived the study, designed, participated in sample collection, performed laboratory experiments, conducted data analysis and drafted the manuscript for publication. AF, TT, KM reviewed the initial and final drafts of the manuscript. ED treated the patients, reviewed the initial and final drafts of the manuscript. All authors read and approved the final manuscript.
